# Beyond the surface: analyzing etomidate and propofol as anesthetic agents in electroconvulsive therapy—A systematic review and meta-analysis of seizure duration outcomes

**DOI:** 10.3389/fneur.2023.1251882

**Published:** 2023-10-17

**Authors:** Syed M. M. Akhtar, Syed Z. Saleem, Syed H. A. Rizvi, Sandesh Raja, Muhammad Sohaib Asghar

**Affiliations:** ^1^Department of Medicine, Dow Medical College, Dow University of Health Sciences, Karachi, Pakistan; ^2^Mayo Clinic, Rochester, MN, United States

**Keywords:** electroconvulsive therapy, etomidate, propofol, seizure duration, meta-analysis

## Abstract

**Background:**

Electroconvulsive therapy (ECT) is a widely used treatment for severe psychiatric disorders such as schizophrenia, depression, and mania. The procedure involves applying brief electrical stimulation to induce a seizure, and anesthesia is used to ensure sedation and muscle relaxation. Finding the right anesthetic agent with minimal side effects, especially on seizure duration, is crucial for optimal outcomes because seizure duration is an important factor in the effectiveness of ECT, but the anesthetic agents used can affect it.

**Objective:**

This systematic review and meta-analysis aimed to pool the results of all relevant studies comparing the two induction agents, etomidate and propofol, for motor and electroencephalogram (EEG) seizure duration outcomes.

**Methods:**

A comprehensive literature search was conducted in the PubMed, Medline, and Cochrane Library databases to identify the relevant articles. The primary outcome measures were motor and EEG seizure durations. Statistical power was ensured by performing heterogeneity, publication bias, sensitivity analysis, and subgroup analysis. Standard mean difference and 95% confidence intervals were calculated for continuous outcomes, and a random-effects model was used.

**Results:**

A total of 16 studies were included in this meta-analysis, comprising 7 randomized control trials (RCTs), 7 crossover trials, and 2 cohorts. The overall motor seizure duration was statistically significantly longer with etomidate than with propofol. The overall result for EEG seizure duration was also longer with the use of etomidate over propofol and was statistically significant. In addition, subgrouping was performed based on the study design for both outcomes, which showed insignificant results in the cohort's subgroup for both outcomes, while the RCTs and crossover subgroups supported the overall results. Heterogeneity was assessed through subgrouping and sensitivity analysis.

**Conclusion:**

Our meta-analysis found that etomidate is superior to propofol in terms of motor and EEG seizure duration in ECT, implying potentially better efficacy. Hence, etomidate should be considered the preferred induction agent in ECT, but larger studies are needed to further validate our findings.

## Introduction

Electroconvulsive therapy (ECT) is a well-established therapeutic intervention employed in the management of acute and severe psychiatric disorders, such as depression due to major depressive disorder or bipolar disorder, mania, schizoaffective disorder, catatonic symptoms, and acute suicidal ideations as these conditions often exhibit resistance to conventional medical treatments (1, 2).

Acute disorders, such as depressive stupor and severe excitement like delirious mania, malignant catatonia, and neuroleptic malignant syndrome, can be treated with ECT as a first-line treatment since it has shown quick relief in symptoms. ECT was first introduced for the treatment of schizophrenia, but since the advent of effective antipsychotic pharmacotherapy, its application has been reduced to be used as an additional treatment option with antipsychotic medication such as clozapine in treatment-resistant schizophrenia (3). ECT stands as the most efficacious and safest treatment option for elderly individuals, particularly those who are unable to tolerate medication regimens due to side effects or comorbidities or experience further deterioration of depressive symptoms with anti-depressive pharmacotherapy (4). Furthermore, anecdotal evidence of its effectiveness in treatment-resistant obsessive-compulsive disorder, Tourette's syndrome, epilepsy, and Parkinson's disease has been identified (3). In the United States, approximately 100,000 patients undergo ECT annually, emphasizing its widespread utilization (5). The procedure entails the application of a brief electrical stimulus, inducing a generalized seizure, thereby constituting a biological treatment modality (4). Interestingly, the origins of employing ECT to elicit epileptic seizures date back to 11 April 1938, when it was initially performed without anesthesia for over three decades (6).

Presently, anesthesia is administered during ECT to ensure sedation and muscle relaxation. Nonetheless, a pivotal milestone in this regard involves determining the most effective anesthetic agent, characterized by optimal dosing, rapid onset, ease of administration, short half-life, minimal postoperative drug-related side effects, limited impact on memory, hemodynamic stability, and minimal interference with seizure quality and duration (7–9).

The mechanism of action underlying ECT revolves around inducing a motor seizure and an electroencephalogram (EEG) seizure lasting at least 25 and 40 s, respectively. This procedure is typically conducted three times per week, spanning 6–12 treatments, with subsequent maintenance therapy ranging from weekly to monthly administrations (1). The efficacy of ECT hinges upon the total duration of the induced seizure (10–13), with EEG activity lasting between 25 and 50 s believed to elicit the most favorable overall therapeutic response. Patients who have a seizure duration of less than 15 s or more than 120 s during their first seizure tend to have a less positive response to ECT (14). However, controversy surrounds the validity of this correlation, and a consensus has yet to be reached regarding its significance. Nevertheless, most studies have identified seizure duration as a crucial determinant of treatment effectiveness. It should be noted that the anticonvulsant properties of several anesthetic agents employed in ECT can potentially impact its efficacy (14), necessitating a delicate balance between optimal anesthesia and seizure duration. Commonly utilized anesthetic drugs for ECT include methohexital, ketamine, etomidate, remifentanil, propofol, etc.

To the best of our knowledge, a similar meta-analysis (15) was published in 2014 comparing etomidate with multiple anesthetic drugs in terms of motor and EEG seizure duration, but it included a limited number of studies. Hence, the objective of this meta-analysis is to present the recent evidence, including additional studies specifically comparing the effect of etomidate on motor and EEG seizure duration with propofol as the control group, thereby aiding in the selection of the best possible anesthetic agent for future use in ECT.

## Methods

### Data sources and search

A comprehensive literature search was conducted in the PubMed, Medline, and Cochrane Library databases from inception to April 2023. The following medical subject heading terms and keywords were used for the database searches:

(“etomidate” [MeSH Terms] OR “etomidate” [All Fields] OR “etomidate s” [All Fields]) AND (“propofol” [MeSH Terms] OR “propofol” [All Fields] OR “propofol s” [All Fields]) AND (“electroconvulsive therapy” [MeSH Terms] OR (“electroconvulsive” [All Fields] AND “therapy” [All Fields]) OR “electroconvulsive therapy” [All Fields]).

We applied no language restrictions and included studies in non-English languages, and the relevant data were translated for interpretation using the Google Translate service. The references of relevant literature were carefully checked for potentially eligible studies. Disagreements were resolved through consensus and, when necessary, through arbitration by a third researcher.

### Study selection and eligibility criteria

Studies that met the following criteria were included: patients aged 18 years and older undergoing ECT and receiving etomidate as an intravenous induction agent and propofol in the control group.

Additionally, we conducted a manual examination of the sources cited in published meta-analyses addressing similar subjects. We included studies regardless of language restrictions and utilized the assistance of the Google Translate service.

The primary goal is to evaluate the duration of seizures, including motor seizure duration and EEG seizure duration. We included prospective and retrospective controlled trials, including crossover trials. We excluded case note analysis, non-human studies, phase I clinical trials, case reports, editorials, abstracts, reviews, comments and letters, expert opinions, studies without original data, and duplicate publications.

### Data extraction and quality assessment

Two investigators (SA and SRa) independently extracted the following information from each included study: study characteristics (first author, year of publication, sample size, and study type), participant baseline characteristics, indication for ECT, type of induction agents, dosages, route of administration, and outcomes of interest.

In cases where the data were presented as the median and interquartile range (IQR), we attempted to obtain the mean and standard deviation values from the authors through communication. However, if we did not receive a response, as a final option, we calculated the mean using a validated formula: mean = 2m + a + b/4, where “m” represents the median, and a and “b” represent the 25th and 75th percentiles, respectively (16). To estimate the standard deviation (SD), we used the formula provided by the Cochrane Collaboration: IQR = 1.35 SD (17, 18). The risk of bias in the eligible RCTs and crossover trials was assessed using the “Cochrane collaboration's tools for quality assessment” (19). The following six components were assessed: (1) random sequence generation, (2) allocation concealment, (3) blinding of participants and personnel, (4) blinding of outcome assessment, (5) incomplete outcome data, and (6) selective reporting.

As for the crossover trials, we assessed the studies in four additional domains: (1) appropriate crossover design, (2) random sequence generation, (3) carryover effect, and (4) unbiased data (20). The Newcastle–Ottawa Scale (NOS) (21) (range: 0–9 stars) was used to rate the methodological excellence of each retrospective cohort study. Three categories, namely, selection, comparability, and outcome, were used to grade the studies. A total of ≥5 stars indicated that the quality of the research was relatively high. All items were independently assessed by two investigators (SA and SS). Disagreements were resolved through consensus and, when necessary, through arbitration by a third researcher (SR).

### Statistical analysis

The meta-analysis was carried out based on the guidelines of the Preferred Reporting Items for Systematic Reviews and Meta-Analyses (PRISMA) statements (22). RevMan (version 5.4; Copenhagen: The Nordic Cochrane Centre, The Cochrane Collaboration, 2014) was used for all statistical analyses. The standard mean difference (SMD) with 95% confidence intervals (CIs) was calculated on pooled effects for continuous variables.

To assess potential statistical heterogeneity among trials, the Higgins *I*^2^ statistics and Cochrane's Q test were used. The meta-analysis was conducted using fixed-effect modeling. Subsequently, after assessing the heterogeneity with fixed modeling, the analysis was repeated using random-effects methods. As a result, all the values reported in the present analysis are derived from random-effect modeling. We measured the degree of heterogeneity among the trials using the *I*^2^ statistics. *I*^2^-values below 40% were deemed insignificant, while those ranging from 30 to 60% indicated moderate heterogeneity. High heterogeneity was represented by values between 50 and 90%, and values exceeding 75% denoted substantial heterogeneity. When the heterogeneity was high, subgroup analysis or sensitivity analysis was used to identify sources of heterogeneity. The results of the meta-analysis were visually examined by a forest plot, and the potential publication bias was shown by a funnel plot and Egger's test. Egger's test was performed using the R statistical software (23) and meta package v4.17-0 (24). A *p*-value of < 0.05 was considered statistically significant.

## Results

### Study characteristics

**Figure 1:**
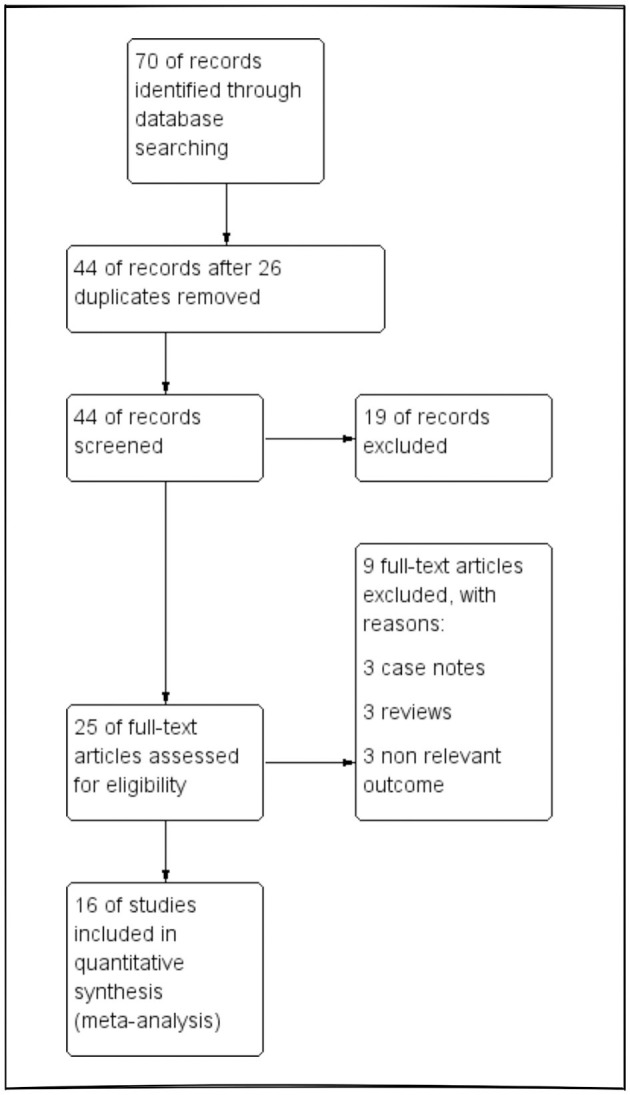
PRISMA flow diagram of the literature search process.

We comprehensively searched PubMed, Medline, and Cochrane Library databases from inception until April 2023 using the keywords “etomidate,” “propofol,” and “electroconvulsive therapy,” and a total of 70 articles were identified. After removing the duplicates, the articles were shortlisted first by the topics, then by reading the abstracts, and finally by way of full-text review. This left us with a total of 16 relevant articles (25–40) that were included in this meta-analysis. A summarized result of our literature search was reported in the PRISMA flowchart (Figure 1).

The baseline characteristics of the included studies are found in Table 1. Out of the included studies, two were of retrospective cohort nature (25, 31), while the rest were clinical trials (27–30, 32, 37, 40), some of which were crossover in nature (26, 33–36, 38, 39). The included articles used etomidate and propofol as interventions. In addition, some studies also included thiopental, thiopentone, ketofol, ketamine, methohexital, and methohexitone as interventions, although we disregarded their results in our analysis. Most of the studies did not mention the indication for ECT; however, those that did usually stated their demographic as depressed (35, 39) or schizophrenic (36, 38). The most reported outcomes of the studies in our analysis included hemodynamic stability (heart rate change, systolic blood pressure change, diastolic blood pressure change, and mean blood pressure), electroconvulsive seizure motor duration, and seizure EEG duration. The mean age of the patients in the included studies ranged from 30.77 to 49.6 years for etomidate and 31.34 to 62 years for propofol.

**Table 1:** Study characteristics of the included studies.

**Study**	**Study location**	**Study design**	**Indication for ECT**	**Groups**	**No. of participants**	**Gender (male/female)**	**Dosage**	**Sedatives used**
Gurel et al. (25)	Turkey	Retrospective cohort	Unspecified	Etomidate Propofol	32 32	29/3 7/25	N/A N/A	Etomidate Propofol Thiopental Ketofol
Mehta et al. (26)	India	Prospective, randomized, crossover study	Unspecified	Etomidate Propofol	30 30	N/A N/A	0.2 mg/kg 1.0 mg/kg	Etomidate Propofol
Rajpurohit et al. (27)	India	Prospective, randomized, double-blind study	Unspecified	Etomidate Propofol	30 30	15/15 16/14	0.1–0.3 mg/kg 1–2 mg/kg	Etomidate Propofol
Jindal et al. (28)	India	Prospective, double-blind, randomized, controlled study	Unspecified	Etomidate Propofol	35 35	13/22 12/23	0.2 mg/kg 1.0 mg/kg	Etomidate Propofol
Mir et al. (29)	India	Randomized, controlled trial	Unspecified	Etomidate Propofol	30 30	14/16 17/13	0.2 mg/kg 1.5 mg/kg	Etomidate Propofol Thiopentone
Canbek et al. (30)	Turkey	Randomized, double-blind, clinical trial	Multiple	Etomidate Propofol	16 20	N/A N/A	0.15–0.25 mg/kg 0.75–1.0 mg/kg	Etomidate Propofol Thiopental
Zahavi et al. (31)	Israel	Retrospective cohort study	Unspecified	Etomidate Propofol	29 23	17/12 3/20	mg/kg 0.6 mg/kg	Etomidate Propofol Thiopental
Wang et al. (32)	China	Randomized, controlled trial	Multiple	Etomidate Propofol	20 20	11/9 9/11	0.3 mg/kg 2.0 mg/kg	Etomidate Propofol
Jun et al. (33)	South Korea	Double-blinded, prospective, randomized, crossover study	Unspecified	Etomidate Propofol	09 09	3/6 3/6	0.15 mg/kg 1.5 mg/kg	Etomidate Propofol Thiopental
Tan and Lee (34)	Malaysia	Prospective, randomized, single-blind, crossover study	Multiple	Etomidate Propofol	10 10	N/A N/A	0.3 mg/kg 1.5 mg/kg	Etomidate IA Propofol IB Propofol group II A Etomidate group II B
Erdil et al. (35)	Turkey	Prospective, randomized, crossover study	Major depression	Etomidate Propofol	14 14	4/10 4/10	0.2 mg/kg 1.0 mg/kg	Etomidate Propofol
Gábor et al. (36)	Hungary	Prospective, randomized, crossover study	Schizophrenia	Etomidate Propofol	30 30	14/16 14/16	0.2 mg/kg 1.0 mg/kg	Etomidate Propofol
Grati et al. (37)	Tunisia	Prospective, randomized, controlled study	Multiple	Etomidate Propofol	12 13	N/A N/A	0.15 mg/kg 1.5 mg/kg	Etomidate Propofol
Gazdag et al. (38)	Hungary	Randomized, crossover study	Schizophrenia	Etomidate Propofol	34 34	14/20 14/20	0.2 mg/kg 1.0 mg/kg	Etomidate Propofol
Avramov et al. (39)	USA	Prospective, randomized, crossover study	Chronic depression	Etomidate Propofol	10 10	5/5 5/5	0.15, 0.2, and 0.3 mg/kg 0.75, 1.0, and 1.5 mg/kg	Etomidate Propofol Methohexital
Zeng-Jun et al. (40)	China	Prospective, randomized with placebo control	Unspecified	Etomidate Propofol	43 39	N/A N/A	0.21–0.3 mg/kg 1.0 mg/kg	Etomidate Propofol Control group

N/A, not available.

### Quality assessment

**Figure 2:**
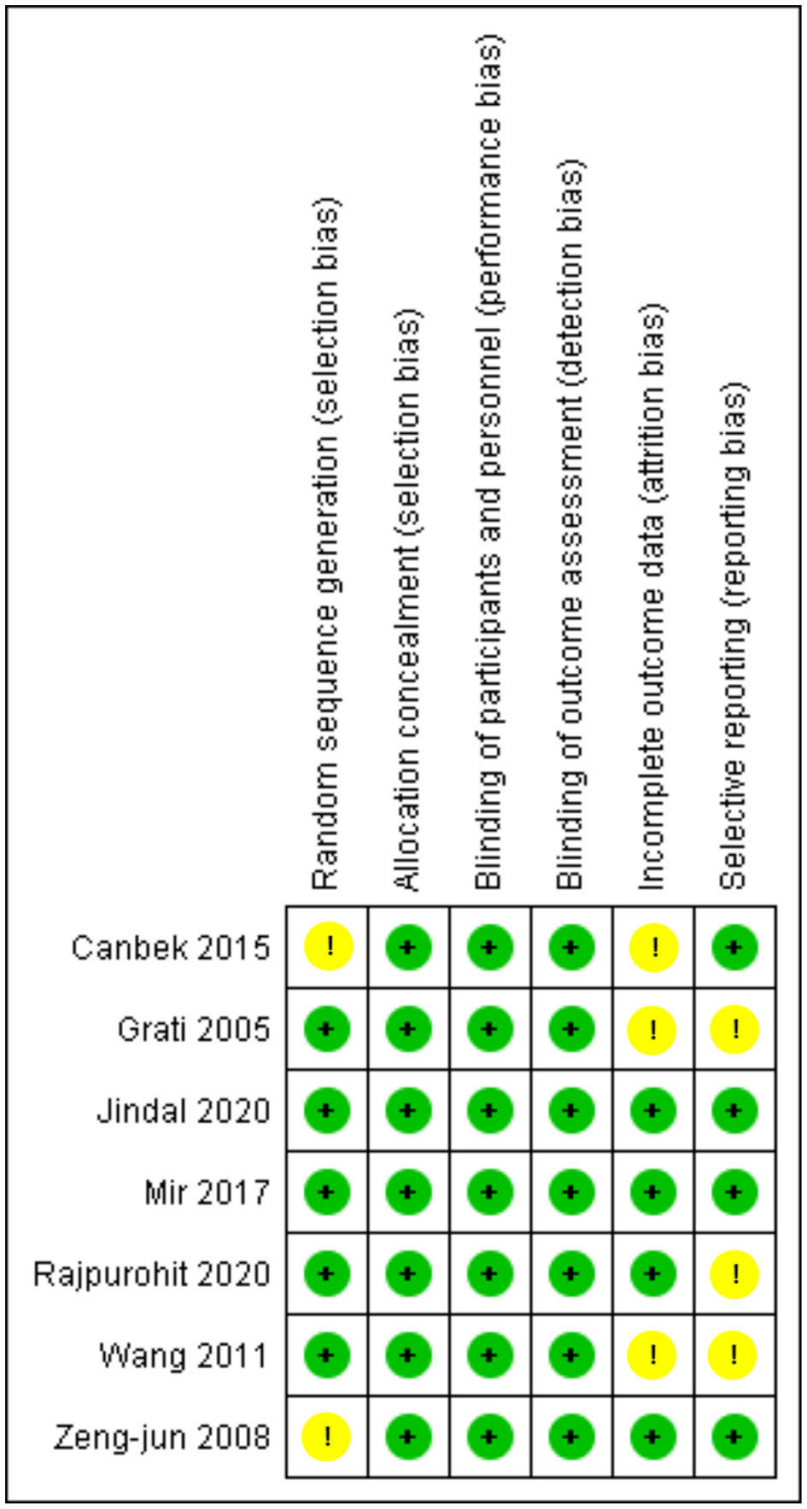
Risk of bias summary for RCTS. The Cochrane “risk of bias” tool was used for quality assessment. Green for “no risk” and yellow for “unclear risk”.

We assessed the quality of the seven RCTs and seven crossover trials using the Cochrane risk of bias tool (19), and overall, the RCTS were found to have a low risk of bias, which are comprehensively shown in Figure 2. Two out of the seven studies were deemed high quality as they had a low risk of bias in all assessing criteria, while the rest of the five studies had an unclear risk mainly in selection, attrition, or reporting bias.

**Figure 3:**
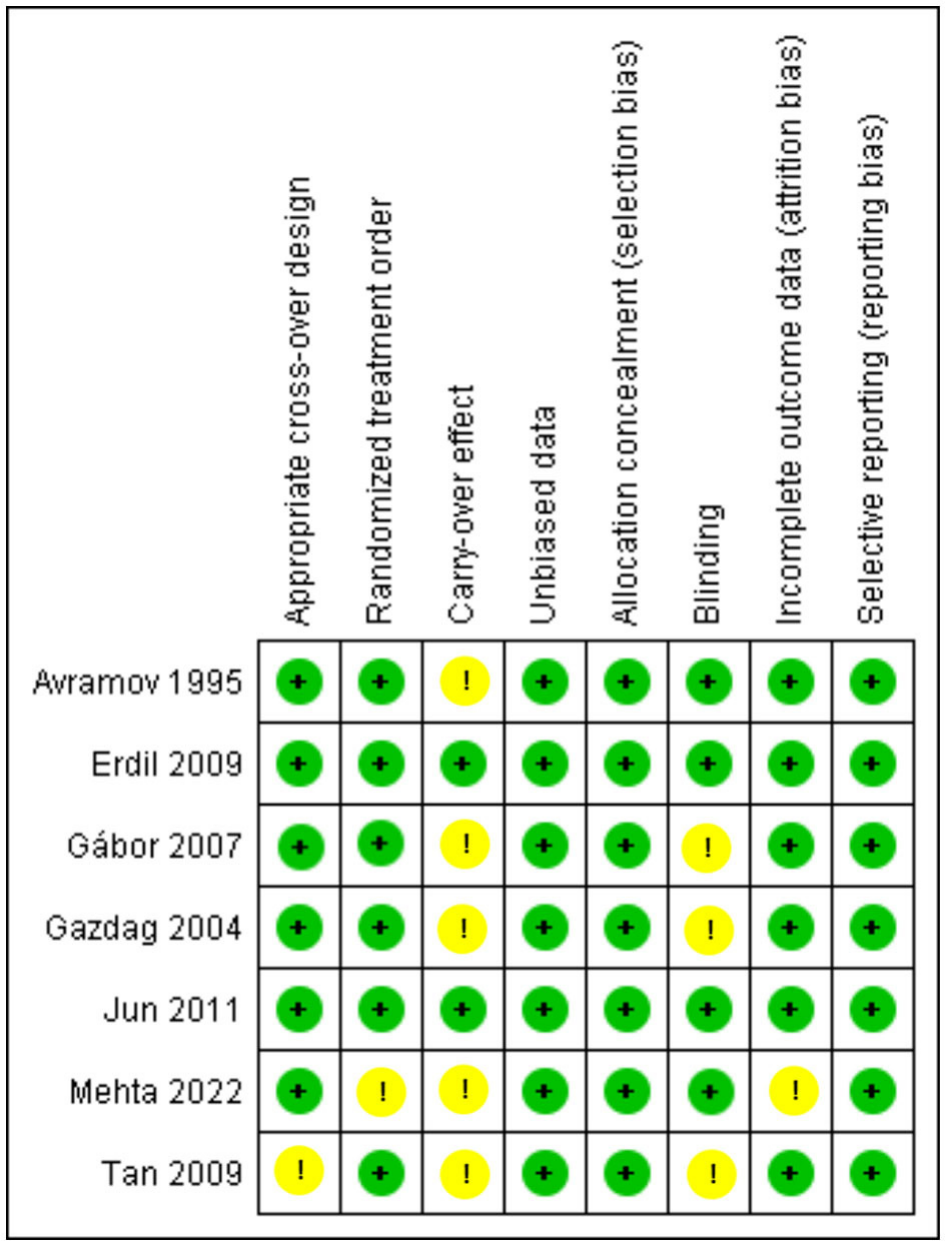
Risk of bias summary for Crossovers: The Cochrane “risk of bias” tool was used for quality assessment. Green for “no risk” and yellow for “unclear risk”.

The crossover studies had a low risk of bias in general, as shown in Figure 3. Two of the studies (33, 35) had a low risk of bias in all assessing criteria and were deemed high quality. The remaining five studies were graded as moderate to good quality as they had problems with only 1 to 2 items of unclear risk. The only two retrospective cohorts (25, 31) included in our study were assessed using NOS (21), and both got 6 and 7 stars, as explained comprehensively in Table 2.

**Table 2:** New Castle–Ottawa Scale for assessment of publication bias of cohort studies.

**Study**	**Selection**	**Comparability**	**Outcome**	**Total**
Gurel et al. (25)	3	2	1	6
Zahavi et al. (31)	4	2	1	7

### Motor seizure duration

**Figure 4:**
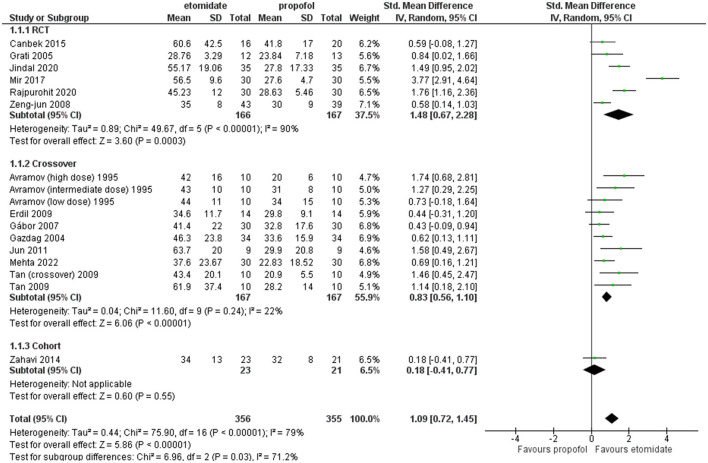
Forest plot of comparison: 1 Etomidate vs. propofol, outcome: 1.1 Seizure motor duration.

All the studies included reported this outcome except these two studies (25, 32). These crossover studies by Tan and Lee (34) divided the study into two phases and performed a crossover, and this study by Avramov et al. (39) classified the patients into three groups of different doses in a double-blind manner; all these results were included in the comparison. Figure 4 shows the analysis of motor seizure duration comparing etomidate and propofol. The overall motor seizure duration with etomidate lasted longer than with propofol (SMD = 1.09, 95% CI = 0.72–1.45; *P* < 0.00001; Figure 4), and the *P*-value (*P* < 0.00001) for this comparison reached statistical significance. The overall heterogeneity (*I*^2^) was high (79%). A subgroup analysis was performed based on study design, i.e., RCTs, crossover, and retrospective cohort studies. In the RCTs and crossover studies subgroup, the results significantly favored etomidate over propofol (SMD = 1.48, 95% CI = 0.67 to 2.28; *P* = 0.0003; Figure 4) and (SMD = 0.83, 95% CI = 0.56–1.10; *P* < 0.00001; Figure 4), respectively. The *I*^2^-values for the comparison were 90% and 22%, respectively. However, the only cohort study showed the difference to be statistically insignificant (*P* = 0.55) (SMD = 0.18, 95% CI = −0.41 to 0.77; Figure 4).

**Figure 5:**
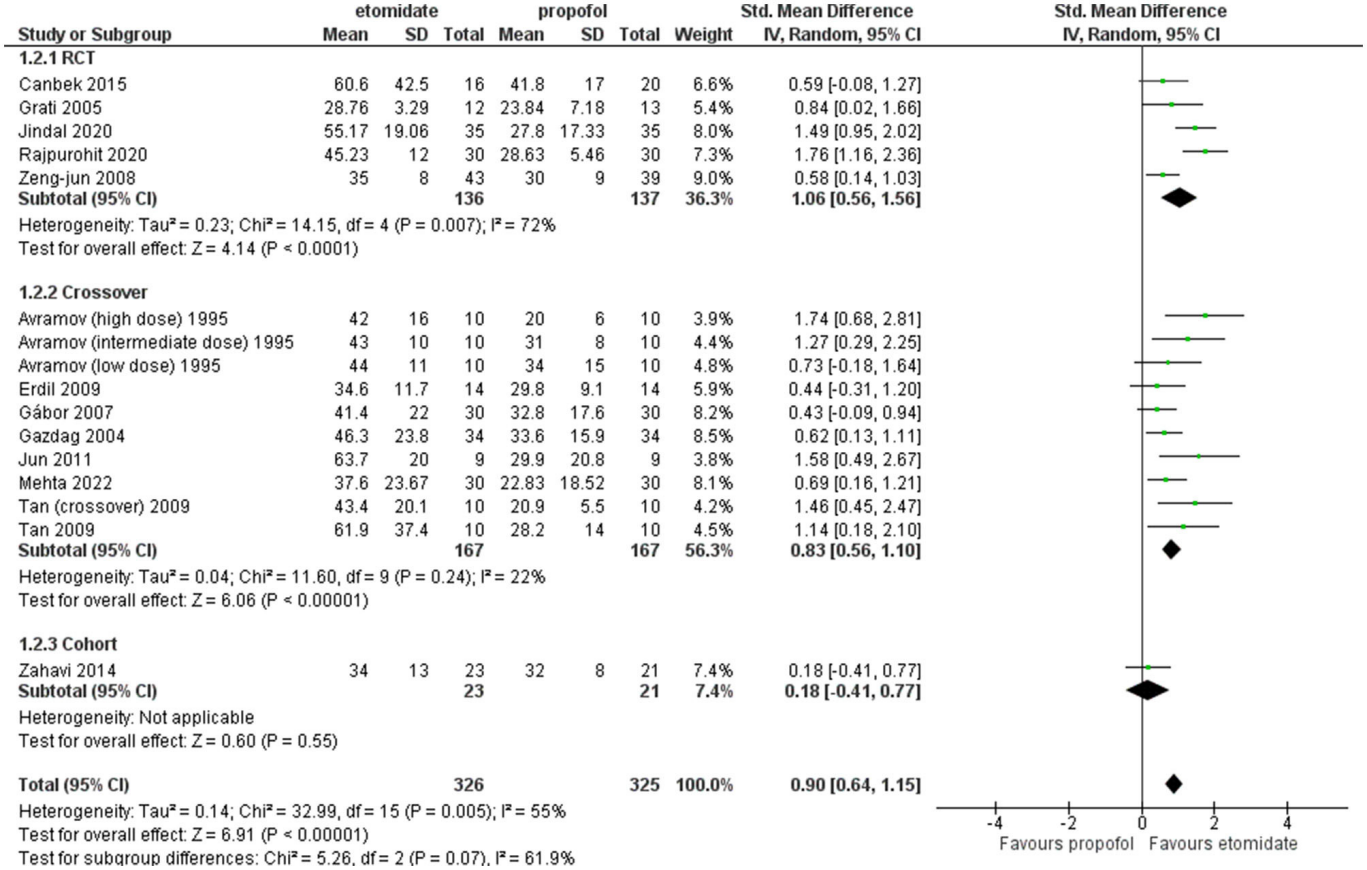
Forest plot of comparison: 1 etomidate vs. propofol, outcome: 1.1 Seizure motor duration [excluding Mir et al. (29)]. Mir et al. was found to be the outlier on sensitivity analysis.

Furthermore, sensitivity analysis revealed this RCT by Mir et al. (29) to be a cause of heterogeneity, and after excluding it, the RCT subgroup and overall result still showed etomidate as having significantly longer seizure motor duration than propofol (SMD = 1.06, 95% CI = 0.56–1.56; *P* < 0.0001; Figure 5) and (SMD = 0.90, 95% CI = 0.64–1.15; *P* < 0.00001; Figure 5), while the heterogeneity lowered down to 72 and 55%, respectively (Figure 5).

### EEG seizure duration

All the included studies reported this outcome except for these five studies (26, 28, 29, 37, 40). Among the crossover studies, Tan and Lee (34) classified the patients into two groups and conducted a crossover design. On the other hand, Avramov et al. (39) classified the patients into three groups, each receiving different doses, in a double-blind manner. The findings from all these studies were incorporated into the comparison.

**Figure 6:**
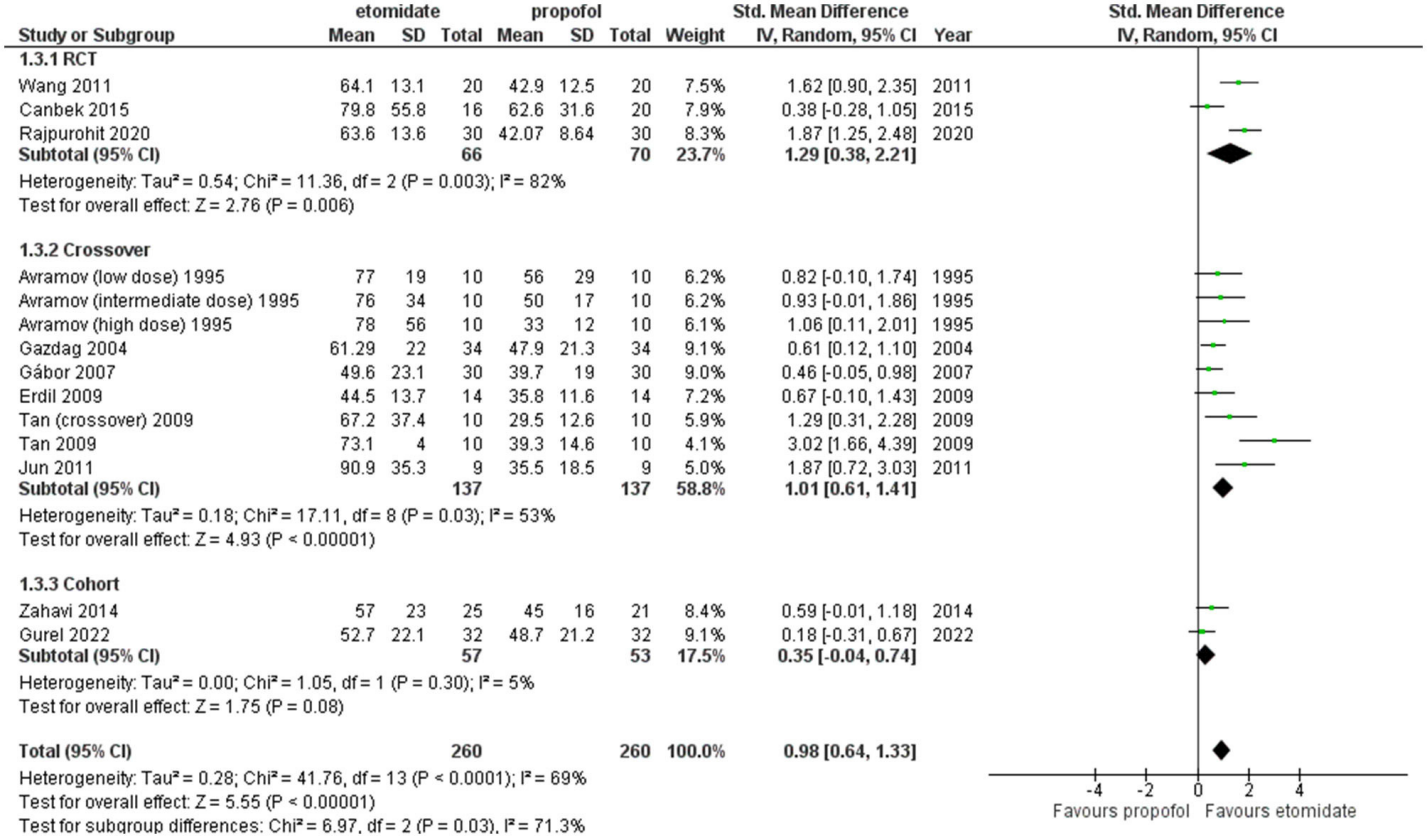
Forest plot of comparison: 1 Etomidate vs. propofol, outcome: 1.2 EEG seizure duration.

The comparison of EEG seizure duration between etomidate and propofol is reported in Figure 6. The overall EEG seizure duration with etomidate lasted statistically significantly longer than with propofol (SMD = 0.98, 95% CI = 0.64–1.33; *P* < 0.00001; Figure 6). The heterogeneity turned out to be 69%. A subgroup analysis was performed based on study design, i.e., RCTs, crossover, and retrospective cohort studies. In the RCTs and crossover studies subgroup, the results significantly favored etomidate over propofol (SMD = 1.29, 95% CI = 0.38–2.21; *P* = 0.02; Figure 6) and (SMD = 1.01, 95% CI = 0.61–1.41; *P* < 0.00001; Figure 6), respectively. The *I*^2^-values for the comparison were 82 and 53%, respectively. While the cohort subgroup showed the difference to be statistically insignificant (*P* = 0.08) (SMD = 0.35, 95% CI = −0.04 to 0.74; *P* = 0.05; Figure 6). The *I*^2^-value for the comparison was 69%.

**Figure 7:**
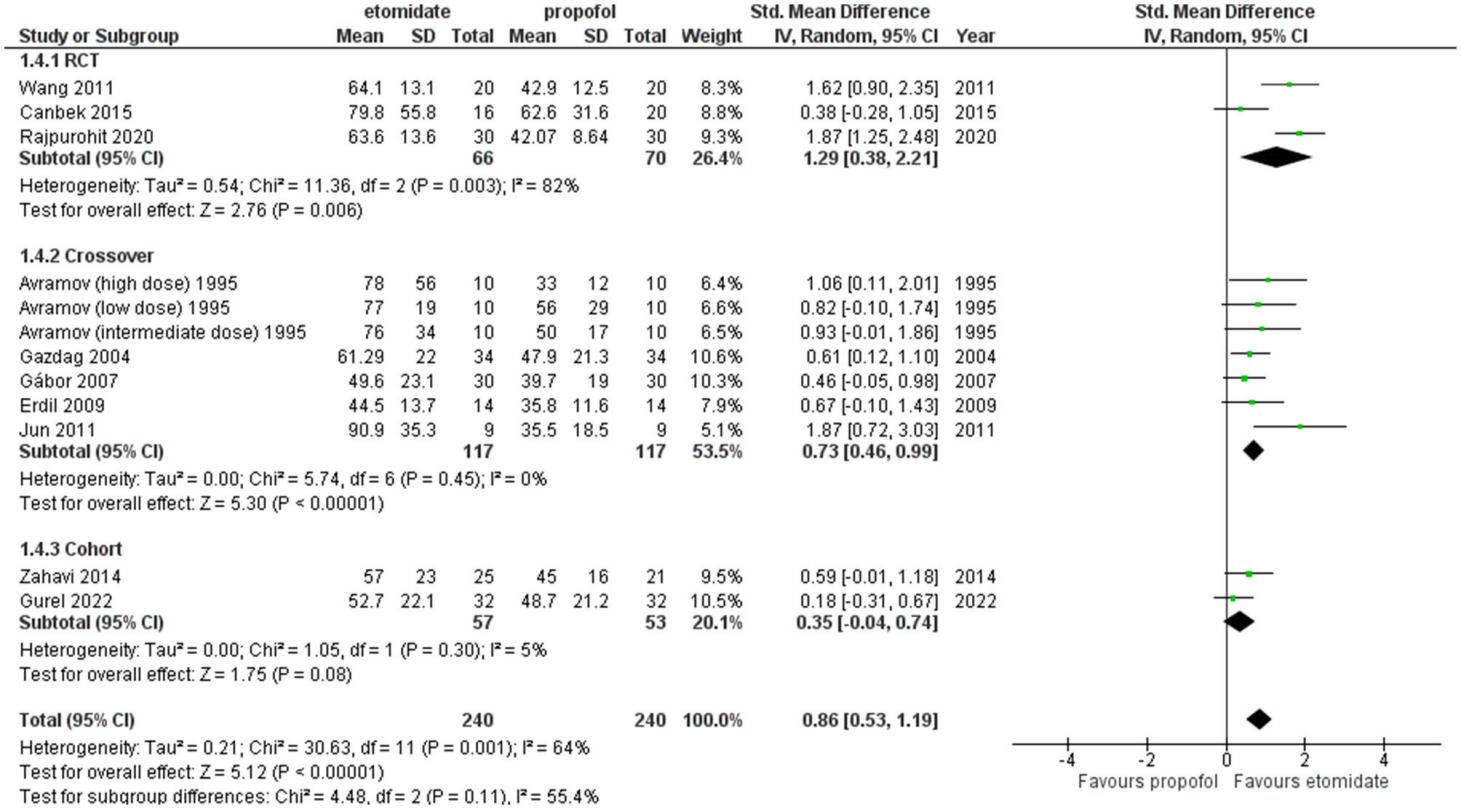
Forest plot of comparison: 1 Etomidate vs. propofol, outcome: 1.2 EEG seizure duration (excluding Tan and Lee). Sensitivity analysis was performed to find out the cause of heterogeneity, and Tan and Lee (34) was found to be the cause.

Furthermore, sensitivity analysis revealed this crossover trial by Tan and Lee (34) to be a cause of heterogeneity, and after excluding it, the pooled result for crossover studies and the overall result turned out to be (SMD = 0.73, 95% CI = 0.46–0.99; *P* < 0.00001; Figure 7) and (SMD = 0.86, 95% CI = 0.53–1.19; *P* < 0.00001; Figure 7) significantly longer with etomidate, while the overall heterogeneity reduced to 64% and between crossover studies completely resolved to 0% (Figure 7).

### Publication bias

**Figure 8:**
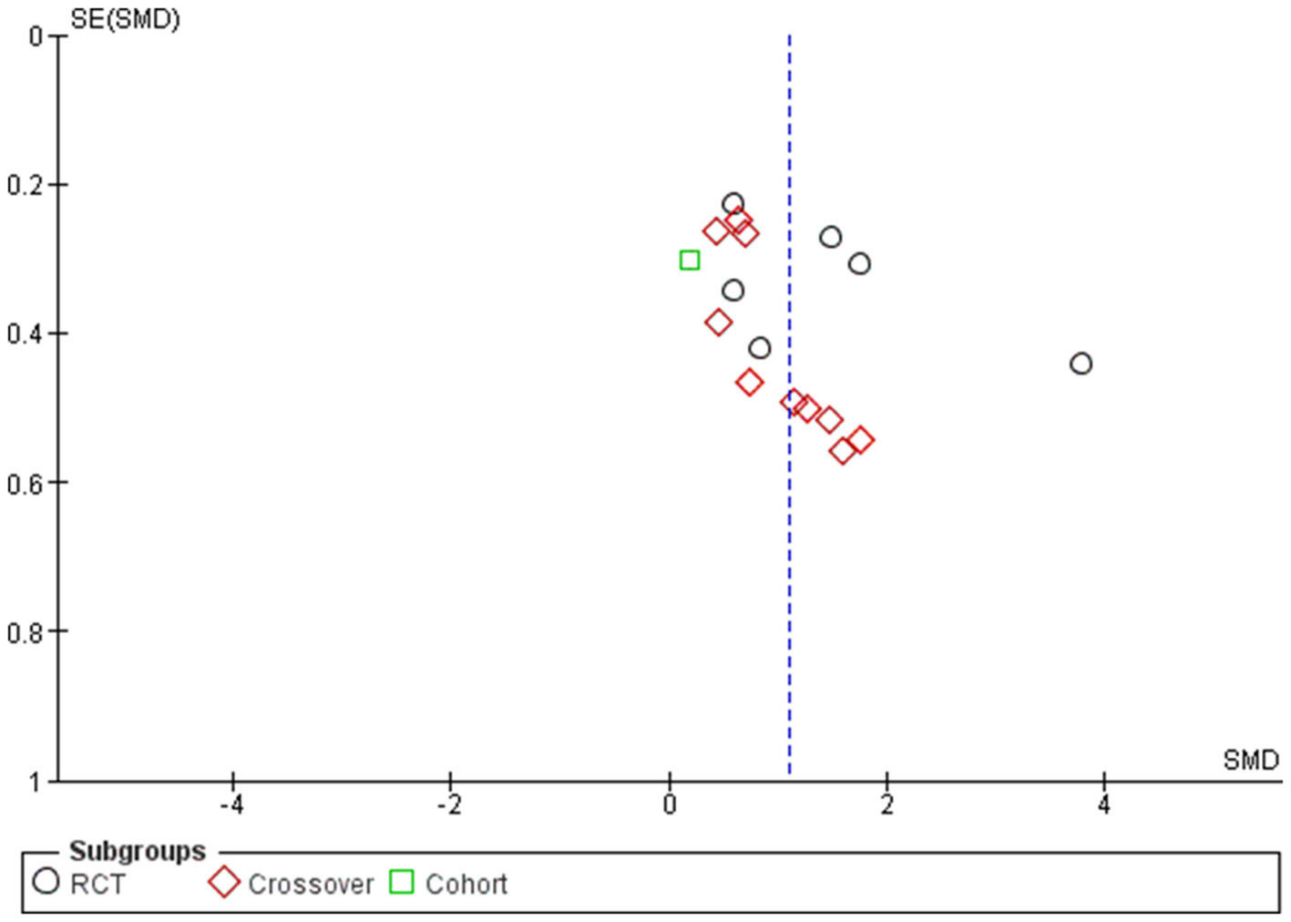
Funnel plot for the outcome of seizure motor duration.

**Figure 9:**
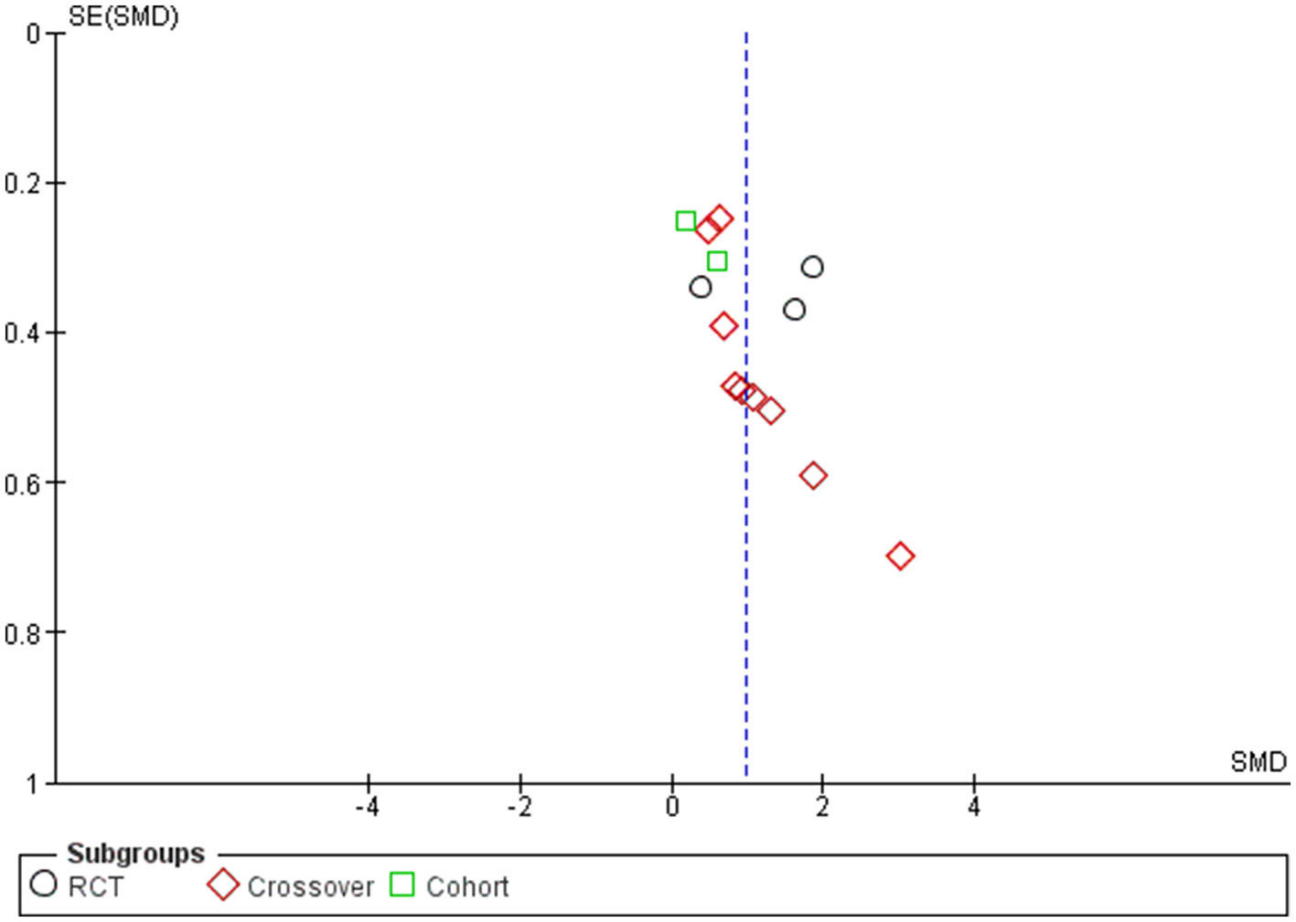
Funnel plot for the outcome of EEG seizure duration.

Two funnel plots for the outcomes of motor seizure duration (Figure 8) and EEG seizure duration (Figure 9) revealed that almost all of the studies shown by scattered points in the funnel plot were distributed in the middle and top of the baseline and were located in the range of the inverted funnel, which implies that there is no significant publication bias and the results from this study are reliable.

Furthermore, to confirm our assessment of publication bias, we performed Egger's test as the number of articles exceeded 10. Egger's test for regression gave an intercept value of 3.227 and a *p*-value of 0.08, indicating no evidence of publication bias for the studies reporting motor seizure duration; however, for EEG seizure duration, Egger's test gave an intercept value of 3.754 and a *p*-value of 0.015, indicating the presence of funnel plot asymmetry and the likelihood of publication bias.

## Discussion

The objective of this meta-analysis was to evaluate these two commonly used sedatives, etomidate and propofol, for motor and EEG seizure duration in ECT. For both the motor seizure duration and EEG seizure duration outcomes, overall, our analysis favored etomidate over propofol as the seizure lasted longer with the use of etomidate, and the results were found to be statistically significant. However, these results should be interpreted with some caution because, as pointed out earlier, seizure duration is a metric used to assess the effectiveness of a seizure, and it should not be automatically assumed that a longer seizure indicates better therapeutic activity. In our analysis, our focus was solely on evaluating seizure duration and not the effectiveness of the anesthetic agents used during induction.

The exact mechanism of action of ECT still remains elusive (41), although some of the possibilities include: (i) facilitation of neurotransmitter release and sensitization of 5-HT1A receptors, which in turn decreases the number of 5-HT2A receptors that are elevated in depressive patients (42); (ii) changes in the sleep architecture due to the seizure provide the therapeutic effect (43); and (iii) ECT seizure induces both anticonvulsant and neurotrophic effects in different parts of the brain (44). EEG and motor seizure duration are a few of the parameters of ECT that are used to judge the efficacy of the procedure as there are some studies that support this correlation (45); however, this remains controversial as there are studies that support the counter-narrative (46).

There are multitudes of different anesthetic agents used to induce anesthesia during ECT. These drugs are known to influence parameters such as seizure duration and hemodynamic stability, which can influence the effectiveness of the therapy and bring about adverse outcomes. Hence, it is imperative that we find an ideal drug that does not hinder the efficacy of ECT. We compared etomidate and propofol because they are widely used. Our analysis concluded that etomidate brings about a significantly longer EEG seizure and motor seizure duration; therefore, it should be preferred over propofol, especially in patients with underlying cardiovascular problems, as etomidate is a much better agent than propofol when it comes to maintaining the hemodynamic stability of the subjects undergoing ECT (26, 35, 38). However, even though potentially more effective, etomidate has its own potential risks as it has been reported to be associated with transient adrenocortical suppression and a risk of increased mortality, especially in patients with sepsis as it inhibits the adrenal mitochondrial 11-β hydroxylase enzyme, which is involved in the conversion of 11-deoxycortisol to cortisol (47). In addition, it commonly exhibits side effects such as myoclonus, plausibly due to the disinhibition of cortical activity (48), which can be decreased via administration of 0.1 mg fentanyl preinduction (49) and carries a risk for pediatric neurotoxicity (50). Furthermore, it is associated with pain at injection sites (34) and increased incidences of nausea and vomiting as compared to propofol, but the occurrences are comparable to general anesthesia (51).

We observed significant heterogeneity between studies in the pooled results for both motor and EEG seizure duration outcomes, potentially due to methodological variations and inadequate blinding among the included studies, which encompassed both retrospective and prospective designs and inconsistent documentation of potential moderator variables, including sex, diagnosis, number of ECT sessions, and duration of disease, as well as ECT technique and dosage variance across the trials. Hence, a subgroup analysis to investigate the observed differences among the trials was performed for both outcomes based on study designs, i.e., RCTs, prospective crossover studies, and retrospective cohorts. For both the motor and EEG seizure duration outcomes, the RCTs and crossover study subgroups significantly favored etomidate over propofol, while even though the cohort study subgroups did favor etomidate, the results were statistically insignificant. In general, an additional subgroup analysis helped in identifying the source of heterogeneity, and to further address this, we conducted sensitivity analysis by removing studies in a stepwise fashion. After eliminating the highly contributing study from each pooled result, the heterogeneity somewhat decreased but did not completely resolve. This implicates high interstudy variance.

Moreover, there is a previously published meta-analysis that compares etomidate with propofol, methohexital, and thiopental (47). It also analyzed the EEG and motor seizure duration and reached the same significant results as we did. It is of note that our analysis included more articles, so it has more power and reports more reliable results.

Finally, our meta-analysis stands out as a comprehensive study encompassing a total of 16 studies, including 7 RCTs, 7 crossover trials, and 2 cohort studies, including a wide range of indications for ECT. We performed a subgroup analysis based on the study design, which was not conducted previously, adding additional merit to our study. The inclusion of both RCTs and retrospective studies further adds to the strength of our findings and provides valuable clinical insights.

### Limitations

Although the findings from our study significantly favor the use of etomidate as the primary drug for ECT, it is important to acknowledge the limitations of our study and exercise caution when interpreting the results.

First, a meta-regression could have been performed as the number of studies exceeded (10) the recommended threshold of 10, as advised by Cochrane (52). This would have allowed for a more in-depth analysis of the variability between studies. Even after performing subgroup and sensitivity analyses, we still detected significant heterogeneity among the studies in the comparison, which we could not eliminate.

Second, the seizure duration can be significantly affected by the dosage of the agent used. Unfortunately, a wide range of doses were employed across all the trials, potentially contributing to the high heterogeneity. Subgroup analysis could have been conducted, but there was a lack of sufficient data. Third, crossover trials, such as the one conducted by Tan and Lee (34), might have influenced the seizure duration outcomes of the agent used post-crossover. However, there is no statistical means of evaluating this hypothesis. Additionally, the site of stimulation for ECT may have affected the seizure duration, but due to a deficiency in available data, we were unable to perform a subgroup analysis. Fourth, due to a lack of sufficient data, this meta-analysis did not take into account side effects or potential complications related to the narcotics, whether they were laboratory-related, clinical (e.g., nausea and vomiting), metabolic (effect on adrenal function), or cerebral (myoclonus). Considering these factors generally influences the strength of recommendations for any intervention. Finally, a risk of publication bias was detected when conducting Egger's test for studies pooled together for the EEG seizure duration outcome. Therefore, additional original studies with larger sample sizes and clear indications of variables such as dosage and method of ECT are required to further confirm the findings of our study.

## Conclusion

Our meta-analysis comparing etomidate and propofol impact on motor and EEG seizure duration revealed etomidate to be clearly better than propofol, in accordance with the conclusion of another previously published meta-analysis (47). Hence, etomidate could be considered the preferable induction agent for ECT over propofol. However, considering the heterogeneity associated with the studies in the current analysis, more and larger studies are needed to further confirm this conclusion.

## Data availability statement

The original contributions presented in the study are included in the article/supplementary material, further inquiries can be directed to the corresponding author.

## Author contributions

The literature search and screening, as well as the collection and analysis of data, documenting the figures, interpreting the data, and preparing the manuscript, were all carried out by SA, SS, SHAR, and SR. SA was responsible for the study's design and analysis, as well as writing the discussion and revising the manuscript. MA provided critical input and drafted the submission. All authors participated in formulating and designing the study and read and gave consent for the final version of the manuscript.
